# Multisystemic Inflammatory Syndrome in Children From the South of Colombia: One Year of Experience

**DOI:** 10.3389/fped.2022.838922

**Published:** 2022-04-05

**Authors:** Pilar Pérez López, Darling Carvajal Duque, Doris Martha Salgado García, Martha Rocío Vega Vega, Jorge Andrés Ramos-Castaneda, Iván José Ardila Gómez, Andrés Felipe Romero

**Affiliations:** ^1^Department of Pediatrics, Hospital Universitario Hernando Moncaleano Perdomo, Neiva, Colombia; ^2^Department of Pediatrics, Clínica Uros, Neiva, Colombia; ^3^Department of Pediatrics, Universidad Surcolombiana, Neiva, Colombia; ^4^Research Group Innovación y Cuidado, Faculty of Nursing, Universidad Antonio Nariño, Neiva, Colombia

**Keywords:** SARS-CoV-2, pediatric multisystem inflammatory disease, COVID-19 related, pediatrics, COVID-19, intensive care units, pediatric

## Abstract

**Background:**

Multisystemic inflammatory syndrome in children (MIS-C) is one of the most severe presentations of COVID-19 infection in pediatrics. Currently, we have few studies that describe the characteristics of this condition in Colombian children.

**Objective:**

To describe the clinical and epidemiological characteristics of children hospitalized with MIS-C in Neiva, Colombia.

**Methods:**

Observational follow-up study of a cohort of children with MIS-C for 12 months (May 15, 2020, to May 30, 2021) in two hospitals in the city of Neiva. Epidemiological data, clinical characteristics, laboratory characteristics, cardiological evaluation, treatment, and clinical outcomes were analyzed.

**Results:**

We included 34 patients who met the diagnosis of MIS-C. The median age was 68 months. Some type of nutritional issue was observed by 43.75% of those under 5 years of age and by 27.78% of those over 5 years of age. Gastrointestinal symptoms were the most frequent, with vomiting, diarrhea, and abdominal pain being the most frequent by 79, 70, and 67%, respectively. By 77% of the patients, a history of SARS-COV-2 infection was documented through IgG. In the echocardiogram, 35.4% of the patients had systolic dysfunction, followed by coronary involvement by 35%.

**Conclusion:**

This study describes a series of cases of children with MIS-C in Colombia. Gastrointestinal manifestations were predominant. Mortality was high in comparison to other countries but similar to that reported in Colombia. This fact was associated with relevant pathological background. More training is required for physicians in order to have a better understanding of the disease so as to have an early diagnosis and timely treatment.

## Introduction

At the beginning of the Severe Acute Respiratory Syndrome Coronavirus 2 (SARS-CoV-2) pandemic, the severity in patients under 18 years of age was underestimated; however, since April 2020 there have been some reports of patients having a hyperinflammatory state and clinical manifestations similar to Kawasaki disease, toxic shock syndrome, or its variants. These patients required hospitalization in an intensive care unit for hemodynamic support or ventilatory support (1, 2). This was later called multisystemic inflammatory syndrome (MIS-C: multisystemic inflammatory syndrome in children).

To date, it is recognized as a disease different from Kawasaki disease (3), with diagnostic criteria defined by the World Health Organization (WHO), the United States Center for Disease Control (CDC), and the Royal College of Pediatrics and Child Health (4–6).

Three phenotypes of the disease have been described (7): Type 1, the most frequent, characterized by multisystemic involvement-related symptoms with a predominance of shock and very high acute phase reactants; type 2, the least frequent with respiratory involvement and with greater severity; and type 3 with manifestations similar to Kawasaki disease. The overall mortality rate is low, close to 1% (8–10).

Studies worldwide have described the clinical and epidemiological characteristics of children in different regions, with reports in Brazil and Chile, in addition to a study of COVID-19 in children, where 95 cases of MIS-C are described (10–14).

Currently in Colombia, the behavior of the infection by SARS-CoV-2(15) and some cases of the behavior of the MIS-C have been reported, focused on patients who require care in the pediatric intensive care unit (PICU) (16–18).

The objective of this study was to describe the clinical and epidemiological characteristics and mortality of pediatric patients who met the WHO criteria for MIS-C in the South Colombian Region.

## Methodology

### Type of Study and Population

An observational follow-up study was conducted in a prospective cohort of pediatric patients diagnosed with MIS-C. Patients between 1 month and <18 years of age, who were admitted to the PICU between May 15, 2020, and May 30, 2021, in two health institutions in the city of Neiva, Colombia, were included.

For the definition of MIS-C, the criteria established by the WHO were considered, and patients who had fever for more than 3 days and at least two of the following clinical manifestations were included: bilateral non-purulent conjunctivitis or signs of mucocutaneous inflammation, hypotension or shock, and cardiac involvement. We defined cardiac involvement as a ProBNP level >350 pg/ml, troponin level >0.1 ng/ml, or ejection fraction lower than 60%; we defined coronary dilatation as a Z-score from 2.0 to 2.4 and coronary aneurism as a Z-score larger than 2.5. Evidence of coagulopathy was defined as the following: thrombocytopenia as a platelet count <150,000/microliter (mcl), D-dimer > 500 ng/ml, acute gastrointestinal involvement manifested as diarrhea, abdominal pain, or vomiting, or elevated inflammation markers like C-reactive protein (CRP) ≥2 mg/dl, erythrocyte sedimentation rate (ESR) ≥20 mm/hr, or procalcitonin ≥2 ng/ml, as well as evidence of SARS-CoV-2 virus infection (through real-time RT-PCR BD MAX™ System or antigenic test through lateral flow immunoassay), presence of antibodies (IgG or IgM for SARS-CoV-2 through flow lateral immunochromatography), or epidemiological link. The follow-up time of the patient cohort was until discharge from the PICU. We defined nutritional condition according to the WHO recommendations. For children older than 5 years, we defined overweight as a body mass index (BMI) for age more than one standard deviation but less than two standard deviations; we defined obesity as a body mass index (BMI) for age more than two standard deviations. The appropriate BMI for age was defined between −1 and 1 standard deviations, and thinness risk was defined as > −1. For children under 5 years of age, we defined overweight according to the WHO for weight and height as more than two standard deviations above the median and obesity was defined as more than three standard deviations, appropriate weight and height between −1 and 1 standard deviations, risk of acute malnutrition between ≥-2 and <-1 standard deviations, and for weight and height and acute malnutrition < −2 standard deviations.

During the whole study period, the collection of patients was carried out through an active surveillance system, where attendance and residence were reported daily on all patients who could meet all the inclusion criteria. These data were reviewed by a transdisciplinary group made up of infectious diseases, rheumatology, cardiology, and pediatrics. Once the criteria were verified, the patients were included or excluded.

### Dependent Variable and Independent Variables

The main outcome variable to evaluate was in-hospital mortality; cardiovascular findings, defined as cardiac involvement as we have described above, was our secondary outcome evaluated. The independent variables were sociodemographic characteristics, nutritional status assessed through body mass index (according to age), and presence of clinical signs (headache, myalgia, rash, cough, respiratory distress, abdominal pain, diarrhea, vomiting and fever). Information was also gathered about the evolution of platelets, ferritin, D-dimer, fibrinogen, and proBNP, at admission and 24 and 48 h after this. Hematology values, liver enzymes (TGO and TGP), kidney function tests (BUN, albumin), procalcitonin, and liver enzymes were also analyzed in this cohort.

### Statistical Analysis

A univariate analysis was performed using centralization statistical parameters such as the median and dispersion as the interquartile range for the numerical variables. The qualitative variables were analyzed by proportion. Linear regression and analysis of variance were used to compare the biomarkers (platelets, D-dimer, fibrinogen, ferritin, and proBNP) at admission, 24 and 48 h, or discharge (platelets and ferritin). A bivariate analysis was performed to compare the independent variables considering in-hospital mortality. Numerical variables were compared using the Mann–Whitney U statistical test. The odds ratio (OR) with their 95% confidence intervals was calculated with the Wald test and *p*-value with Fisher's exact test. The data analysis was carried out using the RStudio 3.5 program.

### Ethical Considerations

The project was presented and approved by the ethics committee of the participating health institutions. The main researcher signed a confidentiality agreement with the institutions. All authorizations complied with the conventions approved in the Declaration of Helsinki in its latest version.

## Result

During the study period, 34 patients with MIS-C criteria were treated in the PICU in two health institutions in the region of Huila, most of them from Neiva (44%). Median age was 68 months with a maximum of 204 months. Eleven percent of the patients had some important medical history, and some type of nutritional alteration was observed in 43.75% of those under 5 years of age and in 27.78% in those over 5 years of age (Table 1).

**Table 1:** Demographic data.

**Datum**	**Characteristic**	***N* = 34**	**%**
Sex	Male	18	52.9
	Female	16	47.0
Background	Neuromuscular disease	1	2.9
	Congenital heart disease	1	2.9
	Lung disease	1	2.9
	Autoimmune disease	1	2.9
Nutritional condition (weight and height) <5a	Overweight	2	12.50
	Appropriate weight –height relationship	9	56.25
	Risk of acute DNT	2	12.50
	Acute DNT	3	18.75
Nutritional condition (BMI) >5a	Obesity	1	5.56
	Overweight	2	11.11
	Appropriate	13	72.22
	Thinness risk	2	11.11
Age (median, RI) /months	68 (7.25–128)		

*BMI body mass index; DNT malnutrition; RI interquartile range*.

**Figure 1:**
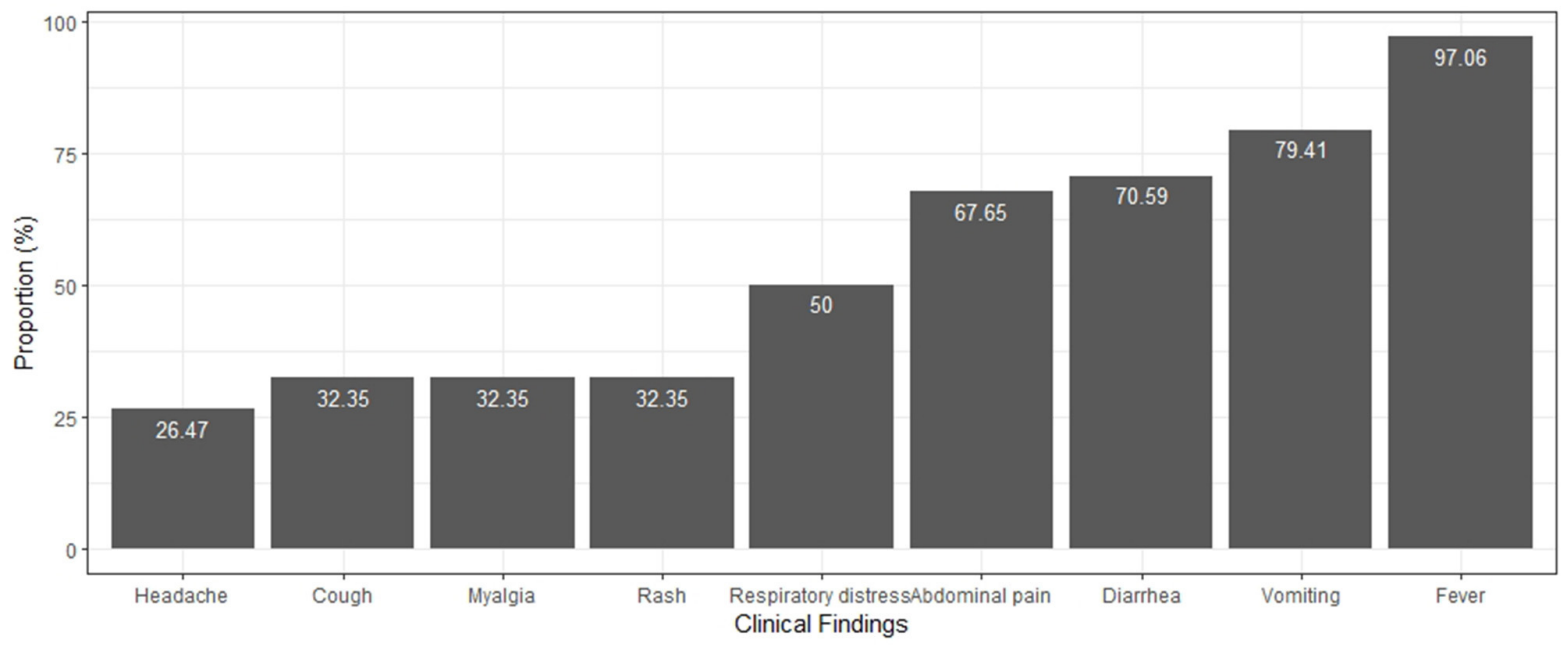
Clinical findings in patients with MIS-C.

Regarding the clinical manifestations, fever was the most frequent in all cases followed by other manifestations in order of frequency as shown in Figure 1. Other signs were observed less frequently, such as aseptic conjunctivitis (17.6%), odynophagia (17.6%), arthralgia (14.7%), lymphadenopathy (11.8%), seizure (11.8%), edema and palmar desquamation (8.82%), and strawberry tongue (2.94%). Eight patients were taken to abdominal surgery, and two had peritonitis. In the evolutionary course, 22 (64%) patients had shock and three died.

In the laboratory findings, no significant abnormalities were documented in the hemogram, clotting times, liver, cardiac enzymes, and renal function tests, but hypoalbuminemia was observed as well as a marked increase in the values of the acute-phase reactants evaluated. The serological study showed positive IgG for SARS-CoV-2 in 77% of the processed samples and IgM only in 27%: regarding the RT-PCR studies, they were carried out in 27 of the patients and the presence of SARS-CoV-2 was shown in half of these cases.

It outstands the fact that in the follow-up, the D-dimer values (median at admission = 2,670.50 ng/ml, median at 48 h = 2,299.0 ng/ml) and proBNP (median at admission = 1,830.0 pg/ml, median at 48 h = 1,442, 5 pg/ml) had a downward trend, while ferritin values increased at discharge, but without statistical significance. A statistically significant difference was only observed in the decrease in fibrinogen (median at admission = 388 mg/dl, median at 48 h = 360 mg/dl, *p* = 0.02).

The cardiac involvement evaluation by means of echocardiography allowed to observe medians of the ejection fractions and shortening of 64 and 33%, respectively, as well as other significant findings shown in Table 2. Coronary involvement was shown in 12 cases (35%), with dilation (Z score 2.0 <2.5) and small aneurysm (Z score 2.5–4.99) being the most frequent (Table 2). Pericardial effusion was shown in 25% of the patients, and less frequent alterations were aortic (*n* = 1) and mitral (*n* = 1) valve disease. One out of three patients developed cardiac arrhythmia, with sinus tachycardia being the most frequent.

**Table 2:** Cardiovascular findings in children with MISC.

**Echocardiogram's findings**	**Median (RI)**	
Ejection fraction% *Median (RI)*	64 (57.25–68.0)	
Shortening fraction% *Median (RI)*	33 (30–37)	
**Other findings**	**Cases (n)**	**%**
Septal dyskinesia *(n)*	9	26.4
Systolic dysfunction *(n)*	12	35.2
Diastolic dysfunction *(n)*	10	29.4
Ventricular dilation *(n)*	6	17.6
Atrial dilation *(n)*	4	11.7
Sinus tachycardia *(n)*	10	29.4
**Coronary involvement** ***(n)***		
Dilation Z score 2–2.4	5	14.7
Aneurysm Z score 2.5–4.99	4	11.76
Aneurysm Z score 5–9.9	2	5.88
Aneurysm Z score >10	1	2.94

*IR, interquartile range*.

In the treatment received, intravenous immunoglobulin G and acetylsalicylic acid (ASA) were used in 29 (85%) and 25 (73%) patients, respectively, becoming the most frequent medication used. Steroids were only used in 10 patients (29%) and always as combination therapy with intravenous immunoglobulin and not as monotherapy. Three out of four patients required respiratory support, with a conventional nasal cannula (11), Venturi (7), and invasive mechanical ventilation (4). 61% of the patients required a vasopressor, 50% received antibiotics, and renal replacement therapy was indicated in 14.71% (*n* = 5), with peritoneal dialysis being the modality used in four children.

When performing the risk analysis comparing deceased patients and survivors, it was found that mortality was associated with cardiovascular findings such as diastolic dysfunction (*p* = 0.02), pericardial effusion (*p* = 0.01), or cardiac arrhythmia (*p* = 0.03) (Table 3).

**Table 3:** Risk analysis in survivors and non-survivor MISC patients.

***Data***	***Non-survivors***	***Survivors***	***p***	***OR (IC95)***
	***N = 3***	***N = 31***		
**Sex**				
Male *n* (%)	1 (33.33)	17 (54.84)	Ref.	1
Female *n* (%)	2 (66.67)	14 (45.16)	0.59	0.41 (0.03–5.03)
**AGE (month)**				
Median (R.I)	11.0 (8.5–10.7.5)	75.0 (12.0–126.0)	1	INF
**Previos medical history**				
Yes *n* (%)	2 (66.67)	2 (6.45)	0.06	22.88 (0.88–1,765)
**Origin**				
Huila *n* (%)	3 (100)	24 (77.42)	0.49	-
**Cardiopulmonary condition** ***n*** **(%)**				
Systolic dysfunction	2 (66.67)	10 (32.26)	0.28	4.2 (0.40–51.98)
Diastolic dysfunction	3 (100)	7 (22.58)	0.02	-
Pericardial effusion	3 (100)	6 (19.35)	0.01	-
Pulmonary hypertension	1 (33.33)	2 (6.45)	0.25	7.25 (0.44–118.70)
Atrial dilation	1 (33.33)	3 (9.68)	0.32	4.67 (0.32–68.03)
Ventricular dilation	2 (66.67)	4 (12.90)	0.07	13.5 (0.98–185.45)
**Ejection fraction**				
Median (r.i)	59.0 (45.0–62.0)	65.0 (57.5–68.5)	0.2	INF
**Shortening fraction**				
Median (r.i)	31.0 (21.0–32.5)	33.0 (30.0–37.0)	0.25	INF
**Dyskinesia**				
Yes *n* (%)	0	9 (29.03)	0.55	-
**Shock**				
Yes *n* (%)	3 (100)	19 (61.29)	0.54	-
**Arrhythmia**				
Yes *n* (%)	3 (100)	8 (25.81)	0.03	-
**Coronary involvement** ***n*** **(%)**				
No	2 (66.67)	18 (58.06)	ref.	1
Yes	1 (33.33)	13 (41.94)	0.82	0.70 (0.02–10.04)
**Leukocytes/mcl**	13,100	14,290		
median (i.r)	(8,345–26,550)	(8,085–19,480)	0.95	INF
**Neutrophils /mcl**	7,700	9,880	0.95	INF
median (i.r)	(4,815–16,150)	(4,456–13,925)		
**Lymphocytes /mcl**	1,220	2,150	0.45	INF
median (i.r)	(950–2,240)	(1,145–3,691)		
**Hemoglobin g/dl**	9.1	11.4	0.25	INF
median (i.r)	(8.85–10.25)	(9.25–1,290)		
**Hematocrit %**	27.8	33.8		
median (i.r)	(27.65–32.55)	(29.25–38.45)	0.4	INF
**ESR mm/h**				
median (i.r)	21.0 (19.50–23.0)	32.0 (22.0–44.0)	0.15	INF
**CRPmg/dl**				
median (i.r)	9.0 (4.75–9.40)	9.0 (3.31–24.72)	0.43	INF
**PCT ng/ml**				
median (i.r)	4.79 (2.44–33.64)	1.47 (0.36–3.59)	0.67	INF
**GOT U/L**				
median (i.r)	109.0 (87.50–3,804.50)	32.10 (23.15–53.0)	0.02	INF
**GPTU/L**				
median (i.r)	32.7 (25.35–2,126.85)	20.0 (12.50–41.50)	0.3	INF
**DHL U/L**				
mediana (i.r)	832.0 (644.0–1,483.0)	296.5 (228.5–360.0)	0.02	INF
**UNB mg/dl**				
median (i.r)	57.0 (33.5–88.5)	9.0 (5.32–12.0)	0.04	INF
**Troponin ng/ml**				
median (i.r)	0.05 (0.04–0.63)	0.15 (0.09–0.33)	0.76	INF
**Albumin g/dl**				
median (i.r)	2.51 (2.21–2.96)	2.91 (2.60–3.32)	0.35	INF

*i.r, interquartile range; OR, odds ratio; GSR, globular sedimentation rate; CRP C, reactive protein; PCT, procalcitonin; GOT, glutamic oxaloacetic transaminase; GPT, glutamic pyruvic transaminase; LDH, lactic dehydrogens; UNB, urea nitrogen in blood, ESR, erythrocyte sedimentation rate; Mcl, microliter; INF, values are infinite*.

The deceased patients had an increase in the median of the TGO (109.0 vs. 32.10 U/l), DHL (832.0 vs. 296.5 U/l), and UNB (57.0 vs. 9.0 mg/dl), being statistically significant (Table 3).

According to the patients' clinical and paraclinical findings in the study, taking into consideration the classification into phenotypes, according to clinical and paraclinical criteria (7), 38% of the patients had MIS-C type 1, 18% of the patients had MIS-C type 2, and 44% of the patients had MIS-C type 3.

## Discussion

MIS-C, considered as a diagnosis of “Novo” in pediatrics, temporarily associated with COVID-19, has been related to a broad spectrum of clinical manifestations, reported in different studies around the world. Here we describe 34 patients with this diagnosis, highlighting within the findings how the cardiovascular system was the most affected, involving 35.4% of cases with systolic dysfunction, followed by coronary involvement in 35%. This proportion of patients suffering from myocardial dysfunction and coronary involvement was higher than that reported in the literature to date, since coronary involvement is described in lower percentages in the different cohorts (19, 20). In Latin American studies such as that in Brazil, this finding was described in 26.5% (14), in Chile in 12% (9). This is likely due to the intrinsic characteristics of the Colombian population, in line with what has been described in studies of countries with ethnic variety, where the Hispanic-Latin or Afro-Caribbean population was the most affected by MIS-C (19, 21). For our thinking, it is possible that cardiac involvement reflects the endothelial damage produced by this condition (22) but also reflects the inexperience of healthcare system response to a new disease, as well as the delays that could exist in healthcare consultation. We also think that this high proportion of coronary compromise and cardiac involvement could be related to the severity of the diseases because all patients required PICU admission; however, Elilarasi et al. (23) reported 68% of coronary compromise, but only 50% of their patients required PICU; this supports the fact that ethnicity could be involved in this outcome.

It was found that the male gender was the most affected in 52.9% of the cases, as well as children older than 5 years, with a 68-month median age of presentation, similar to that described in Europe, America, and Latin America (9, 14, 19, 21). In relation to the patients' nutritional status, Colombia being a middle-high-income country, according to the World Bank classification (24), it was documented that 20% of the cases had some malnutrition, much higher than that reported in Brazil, where this comorbidity is reported in 9.1% of the cases (14) in contrast to that reported by Acevedo et al. (18), in which the patients were obese and overweight by 30% and malnourished by 5.1%.

Regarding the clinical characteristics found in this cohort, fever was documented in all patients, followed by gastrointestinal manifestations and, to a lesser extent, respiratory symptoms as well as mucocutaneous findings, very similar to what has been described in the literature on MIS-C (9, 14, 19–21).

Regarding the demonstration of the hyperinflammatory state in MIS-C (25, 26), the most relevant paraclinical findings were increased erythrocyte sedimentation rate, c-reactive protein and procalcitonin as inflammatory markers, D-Dimer as an inflammation and coagulopathy marker, and proBNP values as a cardiac dysfunction marker (14, 19, 20). The trend of these markers was variable with D-Dimer and proBNP values with a decrease in control at 48 h, which could be correlated with the clinical response to medical treatment.

In 77% of the patients, a history of SARS-COV-2 infection was documented through serology and only 50% of the patients who underwent RT-PCR for SARS-CoV-2 had a positive test. These findings are similar to those reported for Latin America (9, 14) but differ from those reported in the United States where about 99% of the patients had positive IgG-type serology for SARS-CoV-2 (19). This latter is in contrast to what was reported in another Colombian study where 67% of the patients had positive RT-PCR and only 13% had positive serology for SARS-CoV-2 (18).

According to the phenotypic characteristics, the most frequent presentation was MIS-C type 3 with 44% of the patients and the least frequent was type 2. These data do not correlate with that described in the United States, where Type 1 MIS-C was predominant (8).

The most frequent treatment was intravenous human immunoglobulin used in 85% of the cases, followed by the use of ASA (73%). Glucocorticoids were used in 10 patients and always in combination with immunoglobulin; this is similar to what has been reported in different studies worldwide (9, 14, 18). Nowadays, there are no prospective studies evaluating the best therapy, but most of them have shown the effectiveness of the use of immunoglobulin, steroid, or a combination of these in the management of patients with MIS-C (12, 20). Within our cohort, we had 8 patients who required appendectomy, a result that has been published in a preview paper (27).

The average stay in the PICU was 6 days, similar to what was also reported in Colombia and Brazil. In this study, mortality was 8.8%, high in comparison to international cohorts, but similar to what was reported in the Colombian study by Fernández et al., which reached 9% (14, 18).

The inclusion of patients from two centers is a limitation in this study. Although they are a reference for southern Colombia, this is not a representative sample of the country's population. We could not get the exact prevalence of MIS-C patients because we did not have access to the exact data of SARS-CoV-2 infections during the study period because many of the samples were processed by the National Institutes of Health and in some patients we did not have the results back. The main limitation was the low sample size, so it was not possible to perform a stratified analysis and a regression model to identify associated factors. Considering the limitations and understanding of the difficulty in the generalization of the information, this study provides relevant data and analysis of children's behavior with MIS-C in southern Colombia.

COVID-19 vaccination for children has been controversial. Nowadays, we have safety and efficacy data for some vaccine, but we do not know the real impact that children vaccination will have on MIS-C.

## Conclusion

Since the beginning of the SARS-CoV-2 pandemic and the appearance of the first cases of MIS-C, the evidence in the pediatric population has been growing stronger in relation to the clinical affectation, variety of phenotypes, and available therapeutic options. This evidence has shown that a high diagnostic suspicion, active search, and early establishment of treatment, and assigning of a trans-disciplinary team, capable of isolating the needs of each patient, is essential to improving the outcomes and complications in this pathology. It was shown that mortality was related to comorbidities, which has an important implication when considering early vaccination in this population at risk. The long-term sequelae of this disease, especially cardiovascular morbidity, remain to be a great unknown, and it becomes a research topic to understand the long-term behavior of these patients.

## Data Availability Statement

The original contributions presented in the study are included in the article/supplementary material, further inquiries can be directed to the corresponding author.

## Ethics Statement

The studies involving human participants were reviewed and approved by Ethics Committee of the Hospital Universitario Hernando Moncaleano Perdomo–Ethics Committee of the Clinica Uros. Written informed consent was provided by the participants' legal guardian/next of kin to participate in this study in accordance with the national legislation and the institutional requirements.

## Author Contributions

AR and PP designed the entire protocol and were the head of the study. DC, DS, MV, and IA participated in the data collection and the elaboration of the manuscript. JR-C analized the data and participated in the elaboration of the manuscript. All authors contributed to the article.

## Funding

Publication was funded by Universidad Surcolombiana.

## Conflict of Interest

The authors declare that the research was conducted in the absence of any commercial or financial relationships that could be construed as a potential conflict of interest.

## Publisher's Note

All claims expressed in this article are solely those of the authors and do not necessarily represent those of their affiliated organizations, or those of the publisher, the editors and the reviewers. Any product that may be evaluated in this article, or claim that may be made by its manufacturer, is not guaranteed or endorsed by the publisher.
